# Luteolin Relieves Metabolic Dysfunction-Associated Fatty Liver Disease Caused by a High-Fat Diet in Rats Through Modulating the AdipoR1/AMPK/PPARγ Signaling Pathway

**DOI:** 10.3390/ijms26083804

**Published:** 2025-04-17

**Authors:** Pongsakorn Taweesap, Prapassorn Potue, Juthamas Khamseekaew, Metee Iampanichakul, Banyaphon Jan-O, Poungrat Pakdeechote, Putcharawipa Maneesai

**Affiliations:** Department of Physiology, Faculty of Medicine, Khon Kaen University, Khon Kaen 40002, Thailand; pongsakorn.tw@kkumail.com (P.T.); prappo@kku.ac.th (P.P.); juthakh@kku.ac.th (J.K.); meteiam@kku.ac.th (M.I.); banyaphonj@kkumail.com (B.J.-O.); ppoung@kku.ac.th (P.P.)

**Keywords:** luteolin, MAFLD, hepatic steatosis, high-fat diet, AdipoR1, PPARγ

## Abstract

Metabolic dysfunction-associated fatty liver disease (MAFLD) is a significant global public health issue. Luteolin possesses several beneficial biological properties, including antioxidation and anti-inflammation. This study investigated luteolin’s effect and potential mechanisms on MAFLD in high-fat diet (HFD)-fed rats. Rats were administered an HFD supplemented with fructose for 12 weeks to induce MAFLD. After that, the HFD-fed rats were given either luteolin (50 or 100 mg/kg/day) or metformin (100 mg/kg/day) for 4 weeks. Luteolin improved metabolic parameters induced by the HFD, since it decreased body weight, blood pressure, fasting blood glucose, serum insulin, free fatty acids, cholesterol, and triglyceride levels (*p* < 0.05). Luteolin reduced hepatic injury and inflammatory markers in HFD-fed rats (*p* < 0.05). Additionally, HFD-fed rats treated with luteolin showed reduced malondialdehyde and raised catalase activity in plasma (*p* < 0.05). Luteolin attenuated hepatic steatosis compared to the untreated rats (*p* < 0.05). Luteolin also increased plasma adiponectin levels accompanied by upregulation of adiponectin receptor 1 (AdipoR1), AMP-activated protein kinase (AMPK), and peroxisome proliferator-activated receptor γ (PPAR-γ) protein expression in liver (*p* < 0.05). These findings revealed that luteolin ameliorated HFD-induced MAFLD in rats, possibly by reducing metabolic alterations and oxidative stress and restoring AdipoR1, AMPK, and PPARγ protein expression in HFD-fed rats.

## 1. Introduction

Long-term excessive consumption of high-calorie foods, along with a sedentary lifestyle, is the leading cause of positive energy balance and obesity [1]. Additional indicators of metabolic syndrome, such as hypertension, dyslipidemia, insulin resistance, and hyperglycemia, have emerged following prolonged intake of a high-calorie diet. It is known that metabolic syndrome is associated with several health problems such as type 2 diabetes, cardiovascular diseases, and fatty liver [2,3]. Metabolic dysfunction-associated fatty liver disease (MAFLD) is caused by excessive fat accumulation in the liver and is closely linked to metabolic disorders, obesity, type 2 diabetes mellitus (T2DM) [4,5], and cardiovascular diseases (CVDs) [6]. Unlike its previous term, non-alcoholic fatty liver disease (NAFLD), MAFLD diagnosis requires the presence of systemic metabolic dysfunction [7]. The severity of MAFLD has been assessed using fat accumulation, liver histology, and clinical manifestations. Simple steatosis is characterized by fat accumulation over 5% of hepatocytes, and when accompanied by hepatic inflammation, it is referred to as Non-alcoholic steatohepatitis (NASH) [8].

The pathophysiology of MAFLD is multifaceted and linked to insulin resistance in adipose tissue and muscle, which results in increased free fatty acid (FFA) circulation and them being taken up by the liver [9]. In addition, previous publications show that FFAs induce hepatic oxidative stress, which aggravates fatty liver in diet-induced MAFLD in rats [10,11]. A prior study indicates that rats subjected to an high-fat diet (HFD) demonstrate elevated levels of circulating free fatty acids, hepatic steatosis, inflammation, and oxidative stress [12,13]. Hepatic damage, shown by elevated levels of the enzyme alanine aminotransferase (ALT), is seen in HFD-induced liver steatosis rats [14]. A hallmark mechanism in the development of MAFLD is an imbalance of adipokines, adiponectin, and leptin due to adipose tissue malfunction in obesity [15]. It has been found that serum adiponectin levels are low in participants diagnosed with type 2 diabetes [16] and MAFLD [17]. Adiponectin has been proposed to mitigate hyperglycemia, insulin resistance, and diet-induced hepatic steatosis in rats. There is strong evidence supporting that adiponectin enhances insulin sensitivity and fatty acid oxidation, possibly through its downstream adiponectin receptor 1 (AdipoR1), AMP-activated protein kinase (AMPK) [18], and peroxisome proliferator-activated receptor **γ** (PPAR**γ**) [19]. Adiponectin also reduces hepatic lipogenesis and increases fatty acid oxidation via the adipoR1/AMPK pathway [20]. Furthermore, PPARγ is helpful in modulating adipose tissue to augment adiponectin secretion, as elevated adiponectin synthesis is likely a crucial mediator of the systemic effects resulting from PPARγ activation [21].

The primary treatment approach in MAFLD patients is lifestyle modification, including a healthy diet, exercise, and weight loss, while the pharmacological regimens for MAFLD are currently in clinical trials [22,23]. Metformin, a widely used antidiabetic drug, has also been explored for its potential in treating MAFLD [24]. It improves insulin sensitivity, reduces hepatic glucose production, and regulates lipid metabolism through AMPK activation [25]. Studies have shown that metformin can decrease triglyceride accumulation in the liver, prevent oxidative stress, and improve metabolic function in MAFLD models [26].

Luteolin is a naturally occurring flavonoid found in many plants, including fruits, vegetables, and herbs. Studies have demonstrated the potential health benefits of luteolin, which include antioxidant [26,27], anti-cancer [28], and anti-inflammatory [29] properties and improved cardiovascular alterations [30]. It has been discovered that luteolin increases the activity of antioxidant enzymes and decreases signs of oxidative stress in rats that have metabolic syndrome [31]. Additionally, luteolin improves metabolic changes, increases nitric oxide (NO) availability in vascular endothelial cells, lowers reactive oxygen species, and restores normal levels of endothelial nitric oxide synthase (eNOS), superoxide dismutase 1 (SOD1), and microRNA-214-3p expression in obese mice fed with an HFD [30]. Also, luteolin has been shown to reduce insulin resistance, which is a major contributor to MAFLD progression [32]. Therefore, this study aims to investigate the effects and mechanism of luteolin on metabolic parameters, hepatic steatosis, and oxidative stress in an HFD-induced rat model of MAFLD.

## 2. Results

### 2.1. Effect of Luteolin on Body Weight, Organ Weight, and Blood Pressure

**Figure 1:**
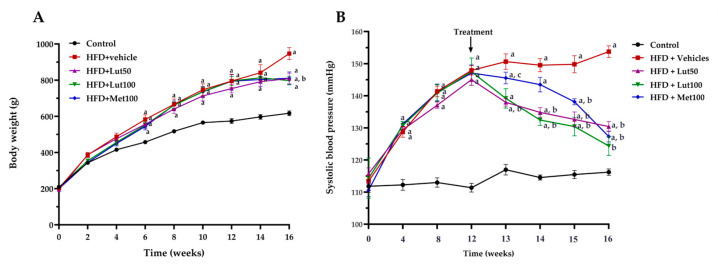
Effect of luteolin on body weight (**A**) and systolic blood pressure (**B**) in all groups of rats. Data are expressed as mean ± SEM. HFD; high-fat diet, Lut50; luteolin (50 mg/kg), Lut100; luteolin (100 mg/kg), Met100; metformin (100 mg/kg). ^a^ *p* < 0.05 vs. control, ^b^ *p* < 0.05 vs. HFD, ^c^ *p* < 0.05 vs. HFD + Lut50 (n = 6/group).

**Figure 2:**
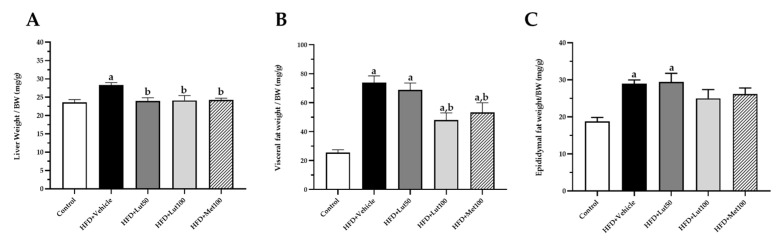
Effect of luteolin on liver weight/body weight (**A**), visceral fat weight/body weight (**B**), and epididymal fat weight/body weight (**C**) in all groups of rats. Data are expressed as mean ± SEM. HFD; high-fat diet, Lut50; luteolin (50 mg/kg), Lut100; luteolin (100 mg/kg), Met100; metformin (100 mg/kg), BW; body weight. ^a^ *p* < 0.05 vs. control, ^b^ *p* < 0.05 vs. HFD, (n = 6/group).

**Figure 3:**
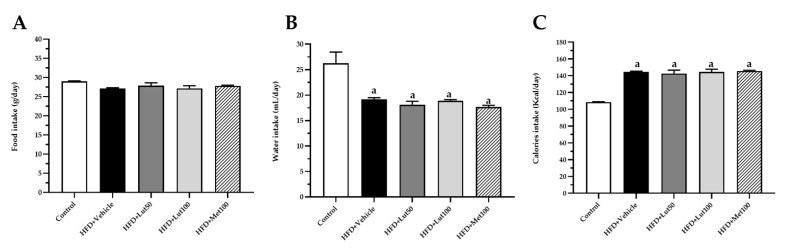
Effect of luteolin on food intake (**A**), water intake (**B**), and calories intake (**C**) in all groups of rats. Data are expressed as mean ± SEM. HFD; high-fat diet, Lut50; luteolin (50 mg/kg), Lut100; luteolin (100 mg/kg), Met100; metformin (100 mg/kg), BW; body weight. ^a^ *p* < 0.05 vs. control, (n = 6/group).

Feeding the rats with an HFD for 16 weeks significantly increased body weight in the HFD-fed group when compared to the control group (*p* < 0.05; Figure 1A). The increase in body weight in the HDF-fed group was consistent with their higher calorie intake, suggesting a relationship between energy consumption and weight gain (*p* < 0.05, Figure 2). In addition, the HFD-fed rats exhibited a higher liver weight, visceral fat weight, and epididymal fat weight (*p* < 0.05; Figure 3) when compared to the control group (*p* < 0.05). The luteolin treatments significantly reduced body weight and organ weight compared to the untreated group (*p* < 0.05). Likewise, the treatment with metformin decreased liver weight, visceral fat weight, and epididymal fat weight compared to the HFD-fed rats (*p* < 0.05). The HFD-fed rats had elevated systolic blood pressure (SP) compared to the normal control rats (*p* < 0.05). Luteolin (50 mg/kg) and metformin (100 mg/kg) significantly reduced SP in the HFD-fed rats (*p* < 0.05) compared to the untreated group. In addition, the value of SP in the HFD-fed rats treated with luteolin (100 mg/kg) was not significant compared to the control rats (*p* < 0.05; Figure 1B).

### 2.2. Effect of Luteolin on Glucose Homeostasis in HFD-Induced MAFLD Rats

The HFD-fed rats had considerably increased fasting blood glucose, fasting serum insulin, Homeostatic Model Assessment for Insulin Resistance (HOMA-IR) index, and area under the curve (AUC) of oral glucose tolerance test (OGTT) compared to the control rats (*p* < 0.05; Table 1). However, the administration of 100 mg/kg luteolin significantly decreased fasting blood glucose, fasting serum insulin, the AUC of OGTT, and the HOMA-IR index (*p* < 0.05; Table 1). This was the same as with the metformin-treated rats compared with the HFD-fed rats (*p* < 0.05; Table 1).

### 2.3. Effect of Luteolin on Lipid Profiles and Inflammatory Parameters

**Figure 4:**
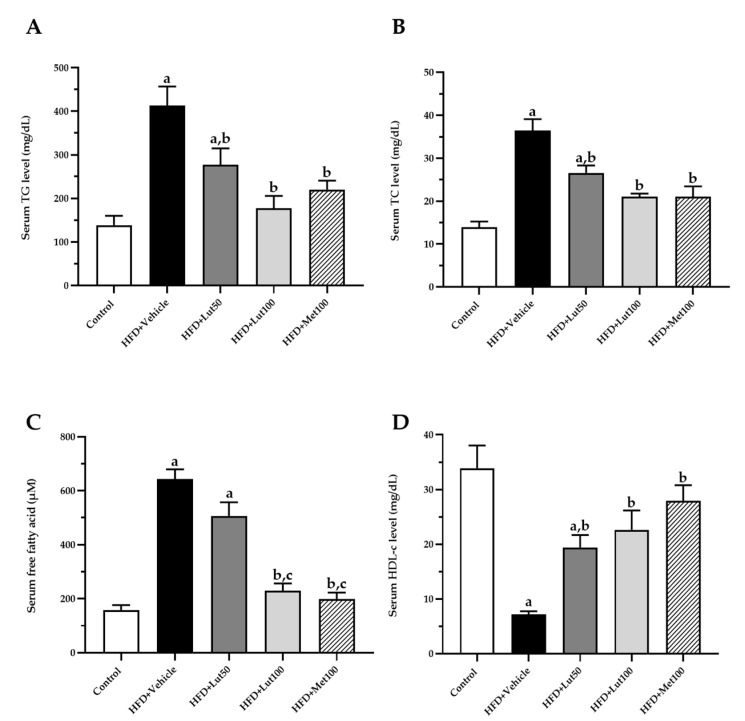
Effect of luteolin treatment on serum TG level (**A**), serum TC level (**B**), serum free fatty acid level (**C**), and serum HDL-c level (**D**) in HFD-induced MAFLD rats. The data are expressed as mean ± SEM. HFD; high-fat diet, Lut50; luteolin (50 mg/kg), Lut100; luteolin (100 mg/kg), Met100; metformin (100 mg/kg), TG; triglyceride, TC; total cholesterol, HDL-c; high-density lipoprotein cholesterol. ^a^ *p* < 0.05 vs. control, ^b^ *p* < 0.05 vs. HFD, ^c^ *p* < 0.05 vs. HFD + Lut50 (n = 6/group).

The levels of total cholesterol (TC), triacylglycerol (TG), and FFA were significantly increased in the HFD-fed rats compared to the control rats (*p* < 0.05; Figure 4A–C). In contrast, the high density lipoprotein cholesterol (HDL-c) level was significantly reduced in the HFD-fed rats when compared to the control rats (*p* < 0.05; Figure 4D). The treatment with luteolin (100 mg/kg) significantly improved the lipid profiles by decreasing TG, TC, and FFA levels while increasing HDL-c levels.

**Figure 5:**
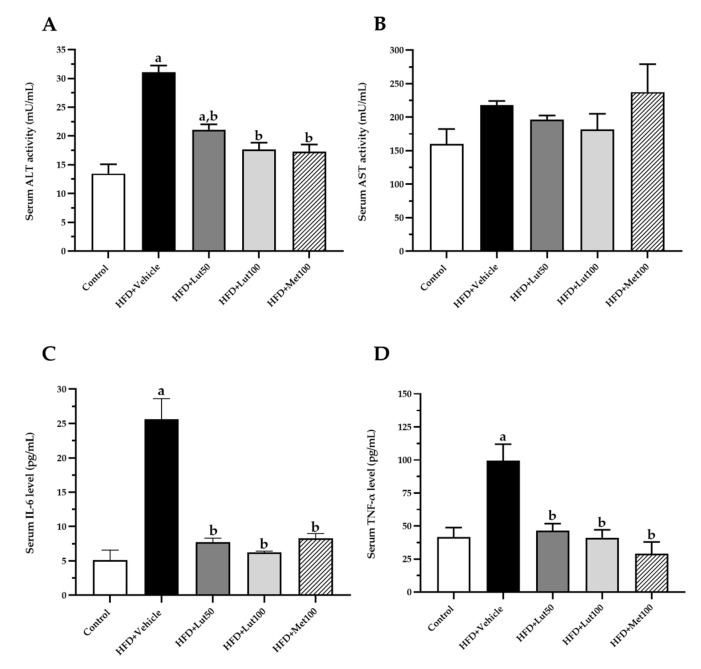
Effect of luteolin treatment on serum ALT activity (**A**), serum AST activity (**B**), serum IL-6 level (**C**), and serum TNF-α level (**D**) on HFD-induced MAFLD rats. The data are expressed as mean ± SEM. HFD; high-fat diet, Lut50; luteolin (50 mg/kg), Lut100; luteolin (100 mg/kg), Met100; metformin (100 mg/kg), ALT; alanine aminotransferase, AST; aspartate transaminase, IL-6; interleukin-6, TNF-α; tumor necrosis factor α. ^a^ *p* < 0.05 vs. control, ^b^ *p* < 0.05 vs. HFD, (n = 6/group).

The concentration of serum ALT activity, serum interlukin-6 (IL-6), and serum tumor necrosis factor-α (TNF-α)were significantly increased in the HFD-fed rats when compared with the control rats (*p* < 0.05; Figure 5). The 100 mg/kg luteolin treatment significantly decreased serum ALT activity, serum IL-6 levels, and TNF-α levels when compared with the HFD-fed rats (*p* < 0.05).

### 2.4. Effect of Luteolin on Oxidative Stress Markers

The levels of plasma malondialdehyde (MDA) and hepatic MDA were significantly increased in the HFD-fed rats when compared to the control rats (*p* < 0.05; Table 2). The production of superoxide (O_2_^•−^) in the liver was significantly raised in the HFD-fed rats when compared to the control rats (*p* < 0.05; Table 2). On the other hand, the HFD-fed rats had significantly reduced catalase (CAT) activity in plasma when compared with the control rats (*p* < 0.05; Table 2). The luteolin and metformin treatment alleviated oxidative status by lowering oxidative stress markers and raising antioxidant activity (*p* < 0.05).

### 2.5. Effect of Luteolin on Histological Examination in Liver

**Figure 6:**
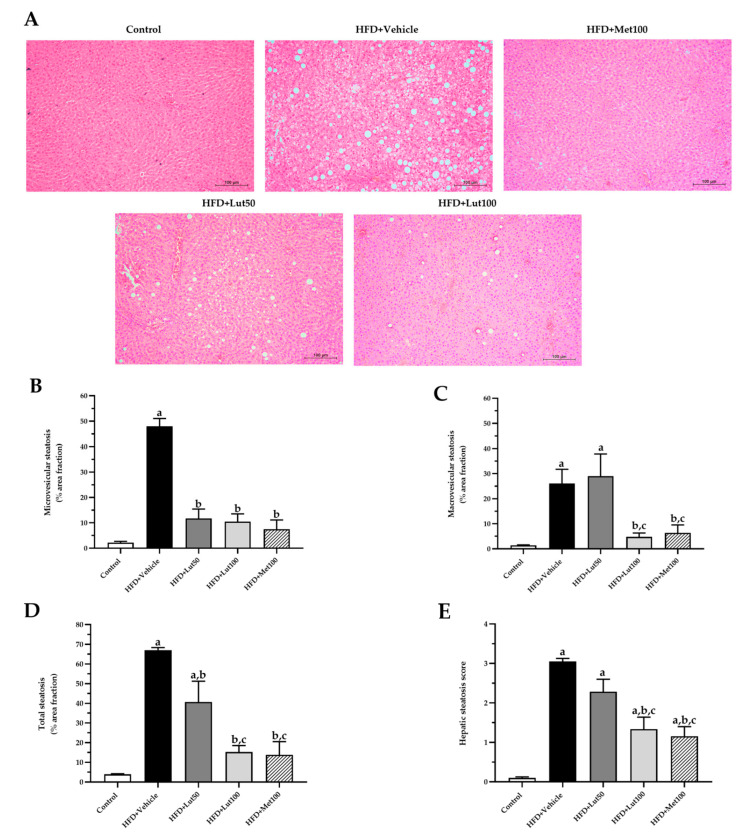
Effect of luteolin treatment on liver histological structure. Representative images of liver sections stained with H&E (**A**), % area fraction of microvesicular (**B**), macrovesicular (**C**), total steatosis (**D**), and hepatic steatosis scores (**E**) in HFD-induced MAFLD rats. The data are expressed as mean ± SEM. HFD; high-fat diet, Lut50; luteolin (50 mg/kg), Lut100; luteolin (100 mg/kg), Met100; metformin (100 mg/kg). ^a^ *p* < 0.05 vs. control, ^b^ *p* < 0.05 vs. HFD, ^c^ *p* < 0.05 vs. HFD + Lut50 (n = 6/group).

Liver morphology was examined using hematoxylin and eosin (H&E)-stained samples from the different experimental groups (Figure 6A). The hepatic steatosis in the HFD-fed rats was markedly elevated in terms of the percentage area fraction of microvesicular, macrovesicular, and total steatosis when compared with the control rats (*p* < 0.05; Figure 6B,D). The luteolin-treated rats (100 mg/kg) had significantly reduced area fraction percentages of microvesicular, macrovesicular, and total steatosis when compared to the untreated group (*p* < 0.05; Figure 6B,D). In addition, metformin helped to reduce these lipid droplets when compared with the HFD-fed rats (*p* < 0.05; Figure 6B,D). Moreover, the hepatic steatosis scores were significantly increased in the HFD-fed rats (*p* < 0.05; Figure 6E). High doses of luteolin significantly decreased the hepatic steatosis scores, similar to the positive control group, metformin, when compared with the HFD-fed rats (*p* < 0.05).

### 2.6. Effect of Luteolin on Adiponectin Levels and AdipoR1 Protein Expression in Liver

**Figure 7:**
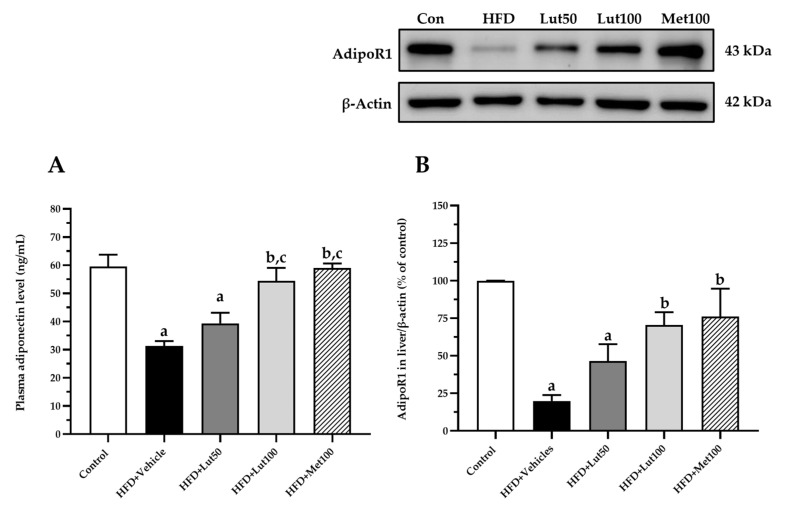
Effect of luteolin treatment on plasma adiponectin level (**A**) and AdipoR1 protein expression (**B**). The data are expressed as mean ± SEM. HFD; high-fat diet, Lut50; luteolin (50 mg/kg), Lut100; luteolin (100 mg/kg), Met100; metformin (100 mg/kg), AdipoR1; adiponectin receptor 1, ^a^ *p* < 0.05 vs. control, ^b^ *p* < 0.05 vs. HFD, ^c^ *p* < 0.05 vs. HFD + Lut50 (n = 6/group).

The levels of plasma adiponectin were reduced in the HFD-fed rats when compared with the control rats (*p* < 0.05; Figure 7A), and the protein expression of AdipoR1 was decreased in the HFD-fed rats when compared with the control rats (*p* < 0.05; Figure 7B). The rats treated with both luteolin and metformin had significantly increased plasma adiponectin levels and upregulated AdipoR1 protein expression in the liver when compared to the HFD-fed rats (*p* < 0.05).

### 2.7. Effect of Luteolin on AMPK and PPARγ Protein Expression in Liver

**Figure 8:**
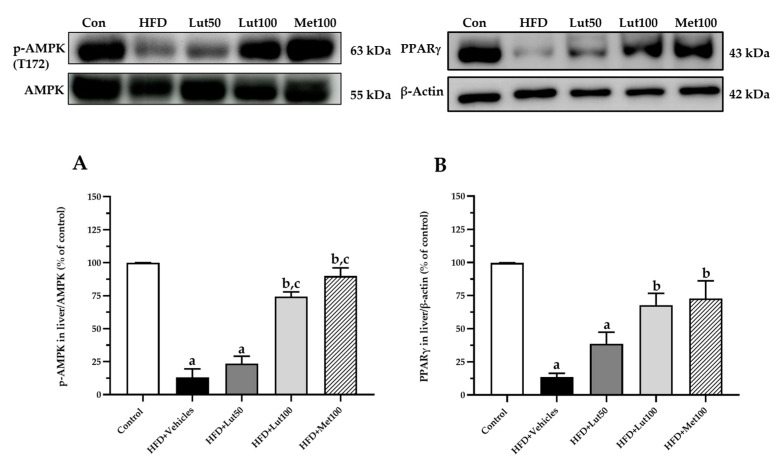
Effect of luteolin treatment on AMPK (**A**) and PPARγ (**B**) protein expression. The data are expressed as mean ± SEM. HFD; high-fat diet, Lut50; luteolin (50 mg/kg), Lut100; luteolin (100 mg/kg), Met100; metformin (100 mg/kg), AMPK; AMP-activated protein kinase, p-AMPK; phosphorylation of AMP-activated protein kinase, PPARγ; peroxisome proliferator-activated receptor gamma, ^a^ *p* < 0.05 vs. control, ^b^ *p* < 0.05 vs. HFD, ^c^ *p* < 0.05 vs. HFD + Lut50 (n = 6/group).

Downregulation of AMPK and PPARγ protein expression was seen in the HFD-fed rats when compared with the control rats (*p* < 0.05; Figure 8). Luteolin (100 mg/kg) or metformin treatment significantly upregulated both AMPK and PPARγ levels when compared to the HFD-fed rats (*p* < 0.05).

## 3. Discussion

The current study revealed that luteolin treatment significantly attenuated HFD-induced MAFLD in rats. Luteolin exhibited dose-dependent effects on lowering metabolic alterations, insulin resistance, oxidative stress, liver damage parameters, and lipid droplet accumulation in the liver of MAFLD rats. Luteolin also modulated adiponectin signaling pathways, as found in the improvements in AdipoR1, AMPK, and PPARγ protein expression in the liver tissue.

We discovered that the rats given an HFD plus fructose exhibited high body and visceral fat weight, SP, insulin insensitivity, hyperglycemia, and dyslipidemia. Our findings support prior studies that created an animal model of metabolic syndrome produced by a high-calorie diet [33]. The obesity in rats found in this study was caused by the high calories of the HFD and fructose, as the food intake was similar between groups. The mechanism of HFD-induced metabolic syndrome involves fat metabolism; once an HFD is digested and absorbed, the circulating FFA levels increase. FFAs are known to promote insulin resistance in target cells, adipose tissue, and skeletal muscle, leading to the dysregulation of glucose and FFA levels [34]. Fructose exacerbates metabolic problems because its metabolism in the liver is unaffected by cellular energy levels and preferentially converts to fat [35]. Hypertension induced by the HFD plus fructose was observed in this experiment. Our observation was in accordance with previous reports [36,37]. In this study, luteolin at a high dose (100 mg/kg) alleviated metabolic abnormalities by reducing body and visceral fat weight, insulin resistance, blood glucose, and dyslipidemia in the HFD-fed rats. Our results were consistent with a previous study that showed that luteolin alleviated lipid metabolism disorders associated with the modulation of the gut microbiota in HFD-fed mice [38].

This study established MAFLD by administering an HFD combined with fructose to rats, resulting in hepatic fat buildup and indicators of metabolic syndrome. Furthermore, ALT levels were elevated in the rats subjected to an HFD, signifying hepatic damage in the current investigation. Oxidative stress was observed in both circulating and hepatic systems in this animal model. Our findings were consistent with previous work that showed that consuming an HFD led to the development of a fatty liver accompanied by high levels of FFAs, oxidative stress, and ALT [12,14]. The mechanism of fatty liver induced by an HFD is known to involve insulin resistance in adipose tissue and skeletal muscle leading to excessive circulating FFAs, which influx to the liver and are stored as TGs [9]. Furthermore, insulin resistance stimulates hepatic de novo lipogenesis by upregulating sterol regulatory element binding protein 1c (SREBP-1c), thereby increasing FFA synthesis and storage in hepatocytes [39]. This excessive lipid accumulation leads to mitochondrial dysfunction and cell death, resulting in ALT leakage into the bloodstream [40]. In this investigation, the HFD-fed rats displayed systemic inflammation due to increased blood TNF-α and IL-6 levels, but there was no evidence of immune infiltration in the hepatic sections. The results, encompassing systemic inflammation and oxidative stress, indicated that simple steatosis was progressing to NASH [41]. Our results aligned with prior publications [42,43]. We demonstrated that fatty liver induced by an HFD was alleviated with luteolin; the underlying processes appear to be its influence on increased insulin sensitivity and oxidative capacity. The effect of luteolin on insulin sensitivity in this investigation was corroborated by recent research demonstrating that luteolin alleviated insulin resistance in metabolic-syndrome-induced cardiac injury rats [31]. Luteolin exerts its hepatoprotective effects by lowering oxidative stress in rats exposed to methamphetamine [44]. Luteolin, with four hydroxyl groups, exhibits potential antioxidant capacity since it reduced lipid peroxidation and enhances the endogenous antioxidant system [45,46]. Luteolin demonstrated anti-inflammatory properties in this investigation by decreasing serum proinflammatory cytokines, aligning with several findings [47,48].

We revealed that the HFD-fed rats had a low level of adiponectin and protein expression of AdipoR1, AMPK, and PPARγ. These abnormalities were recovered after treatment with luteolin. The obesity and increased visceral fat levels caused not just a fatty liver but also adipose tissue malfunction, as seen in the decreased adiponectin levels. Our results agreed with a previous study that showed that adiponectin stimulates its cascade to enhance insulin sensitivity [18,19]. A previous report revealed that adiponectin-induced activation of AdipoR1 stimulates AMPK signaling in the liver, reduces gluconeogenesis, and subsequently improves insulin resistance [49]. Our study found that luteolin increased hepatic PPARγ expression in the HFD-fed rats. This upregulation of PPARγ was caused by the AdipoR1/AMPK signaling pathway, which plays a pivotal role in improving dyslipidemia. PPARγ, which is widely expressed in adipose tissue and the liver, restores lipid metabolism within the liver [50]. This work suggests that luteolin may relieve MAFLD in HFD-fed rats by normalizing the protein expression of the adiponectin/AdipoR1/AMPK/PPARγ pathway.

This study has some limitations, because a study of the pharmacokinetics of luteolin after oral administration was not included. However, a previous systematic pharmacokinetic study revealed that luteolin was rapidly and efficiently absorbed in the rat gut and then substantially metabolized, resulting in a poor bioavailability of 17.5% for unaltered luteolin. Luteolin primarily exists as conjugates in systemic circulation, with luteolin-3′-O-β-D-glucuronide being the most prevalent in plasma and various tissues, including the gastrointestinal tract, liver, kidneys, and lungs [51].

Metformin was used as a positive control agent in this study, and it had a similar effect to the high dose of luteolin, mainly in terms of reducing metabolic alterations and improving insulin resistance. Several publications support our results, indicating that 100 mg/kg of metformin can mitigate various metabolic effects in HFD-fed rats [52,53,54]. Furthermore, the metformin treatment specifically activated AMPK, leading to the downregulation of SREBP-1c and the stimulation of PPARγ expression in the liver. This cascade accelerated adiponectin production, which, in turn, promoted AdipoR1 expression in the liver [19,55,56]. Additionally, metformin also exhibited antioxidant enzyme activity and reduced oxidative stress markers in rats [57].

## 4. Materials and Methods

### 4.1. Chemicals

Luteolin (chemical formula = C_15_H_10_O_6_, molecular weight = 286.24, purity ≥ 95%, chemical abstracts service number = 491-70-3, and catalog number = BP0896) was purchased from Chengdu Biopurify Phytochemicals Ltd. (Chengdu Biopurify Phytochemicals Ltd., Chengdu, China). Metformin was purchased from Siam Pharmaceutical Company Ltd. (Siam Pharmaceutical Company Ltd., Bangkok, Thailand). Other chemicals were obtained from Sigma-Aldrich Corp (Sigma-Aldrich Corp, St Louis, MO, USA).

### 4.2. Animal and Experimental Groups

Thirty male Sprague–Dawley rats (180–200 g) were purchased from Nomura Siam International Co., Ltd., Bangkok, Thailand. The rats were housed at the Northeast Laboratory Animal Center under controlled conditions (23 ± 2 °C and 12 h light/dark cycle). After acclimatization, all rats were divided into five groups (6 rats/group): control, HFD, HFD + luteolin (50 mg/kg/day), HFD + luteolin (100 mg/kg/day), and HFD + metformin (100 mg/kg/day). The vehicle, luteolin, and metformin were administered orally using a gavage tube daily for the last four weeks. The dose of luteolin was chosen based on a previous study [32,58]. In the HFD-fed group, the rats were given an HFD (composed of 24.29% fat, 13.25% protein, and 46.3% carbohydrates) together with 15% fructose water to induce MAFLD in the rats, while the control group was given a standard chow diet (containing 5.72% fat, 22.9 g protein/100 g, and 57.81 g carbohydrates/100 g) and drinking water. Food and water intake were monitored to calculate calorie intake in all groups of rats. In addition, body weight was measured weekly throughout the 16 weeks of study. All the experimental protocols were approved by the Animal Ethics Committee of Khon Kaen University (IACUC-KKU-74/66), Khon Kaen, Thailand.

### 4.3. Systolic Blood Pressure Measurements

Systolic blood pressure (SP) was measured in conscious rats using tail-cuff plethysmography (CODA software version 4.2, non-invasive blood pressure system; Kent Scientific, Torrington, CT, USA). For the twelve weeks of the induction period, SP was measured every two weeks and then every week for the last four weeks of the treatment period. Before the experiment, all rats were trained in a restrainer for one week to acclimatize to the process. The mean values of ten measurements in each rat were recorded. These data were presented as the mean ± SEM.

### 4.4. Glucose Metabolism Determination

Fasting blood glucose was monitored during weeks 12 and 16 using a glucometer. In addition, an OGTT was performed by administering a 20% glucose solution (2 g/kg BW) before measuring the blood glucose levels 0, 30, 60, 120, and 180 min later. Serum insulin levels were measured using ELISA kits (Millipore Corporation, Billerica, MA, USA), and the HOMA-IR was calculated from the following equation: HOMA-IR = fasting glucose × fasting insulin/405.

### 4.5. Lipid Metabolism Determination

Lipid profile parameters, including TG, TC, and HDL-c, were measured using commercial kits (Human Gesellschaft für Biochemical und Diagnostical, Wiesbaden, Germany), while the level of FFA was determined by a quantification assay kit (Abcam, Plc., Cambridge, UK).

### 4.6. Inflammatory Markers, Plasma Aspartate Transaminase, Alanine Aminotransferase, and Adiponectin Analysis

The levels of TNF-α and IL-6 were determined in the serum using commercial kits (ab236712, Abcam, Cambridge, MA, USA, and RE3186RG, AFSBio, Wuhan, China, respectively). In addition, the levels of ALT and aspartate transaminase (AST) were measured using commercial kits (Human Gesellschaft für Biochemical and Diagnostical mbH, Wiesbaden, Germany). Plasma adiponectin levels were assessed using rat ADP/Acrp30 ELISA kits (Reed Biotech, Wuhan, China). The protocol measured was compliant with the guidelines of the kit assay.

### 4.7. Oxidative Stress and Antioxidant Biomarker Determination

Plasma and liver MDA levels were assessed based on the thiobarbituric acid method, as previously described [59]. Using a spectrophotometer, the absorbance of the supernatant was measured at 532 nm. A standard curve was created at various concentrations of 1,1,3,3-tetraethoxypropane, ranging from 0.3 to 10 μmol/L. The CAT activity in plasma was evaluated following a previous method with some modifications [60]. At a wavelength of 405 nm, a yellowish combination of molybdate and H_2_O_2_ was identified, and a standard curve was produced. Plasma CAT activity was measured and is represented in units of U/mL.

The production of O_2_^•−^ in the liver tissue was determined based on the lucigenin-enhanced chemiluminescence method, as described previously [61]. The production of O_2_^•–^ was exhibited as relative light unit counts/minute/dried weight of liver.

### 4.8. Histopathology Analysis

The liver samples were kept in 4% paraformaldehyde for 24 h before being processed and sectioned. Hepatic morphology was evaluated on tissue sections stained with H&E. The level of hepatic steatosis was expressed as the percentage of the area fraction, including microvesicular (small lipid droplets in hepatocytes), macrovesicular (large lipid droplets in hepatocytes), and total hepatic steatosis. This was evaluated by researchers who selected ten random images per number [60]. The hepatic steatosis score was evaluated using a previously described method with some modifications [62]. Briefly, scoring was based on histological criteria, including microvesicular steatosis (score 0–3), macrovesicular steatosis (score 0–3), and hepatocellular hypertrophy (score 0–3). These were categorized as follows: score 0 (<5%), score 1 (5–33%), score 2 (34–66%), and score 3 (>64%). All histological criteria were combined to determine the final hepatic steatosis score.

### 4.9. Western Blot Analysis

The expression of AdipoR1, AMPK, and PPAR*γ* protein in the liver was evaluated by using the Western blot method following a previous description with some modifications [63,64]. This study used specific primary antibodies against AdipoR1 (ab126611, dilution 1:1000, Abcam, Plc, Cambridge, UK), p-AMPK (ab133448, dilution 1:1000), AMPK (ab80039, dilution 1:1000, Abcam, Plc, Cambridge, UK), and PPAR*γ* (sc-27139, dilution 1:1000, Santa Cruz Biotechnology, Inc., Dallas, TX, USA). Signals were captured using an Amersham Imager 600 (GE Healthcare Life Science, Uppsala, Sweden) after being generated with Immobilon Forte Western HRP Substrate (EMD Millipore Corp., Burlington, MA, USA). The intensity of these protein bands was adjusted to that of β-actin and is represented as a percentage relative to the control group from the same gel.

### 4.10. Statistical Analysis

All data are presented as mean ± standard error of the mean (SEM). The Prism 10.0 software (GraphPad Software Inc., LaJolla, CA, USA) was used for statistical analysis. The body weight and SP data were tested using a two-way analysis of variance (ANOVA). When significant differences (*p* < 0.05) were discovered, Tukey’s post hoc analysis was performed. In addition, statistical differences between groups were analyzed using one-way ANOVA followed by Tukey’s post hoc test, with *p*-values < 0.05 considered statistically significant.

## 5. Conclusions

The present study found that luteolin ameliorated MAFLD by reducing metabolic alterations in HFD-fed rats. The molecular mechanism might be related to its antioxidant properties and the modulation of AdipoR1, AMPK, and PPARγ protein expression in the liver tissue. These data provide information regarding the possible role of luteolin as a potential compound for the treatment of MAFLD and offer insights into novel treatment strategies for metabolic liver disease in the future.

## Figures and Tables

**Table 1:** Effect of luteolin treatment on glucose homeostasis in HFD-induced MAFLD rats.

Parameters	Control	HFD + Vehicle	HFD + Lut50	HFD + Lut100	HFD + Met100
Fasting blood glucose (mg/dL)	98.33 ± 0.92	127.17 ± 1.96 ^a^	112.83 ± 2.07 ^a^	99.57 ± 1.46 ^bc^	100.33 ± 3.83 ^bc^
Fasting serum insulin (mg/dL)	103.31 ± 11.64	260.21 ± 26.94 ^a^	190.04 ± 32.63	161.47 ± 14.72 ^b^	163.01 ± 17.55 ^b^
HOMA-IR index	23.82 ± 1.18	74.58 ± 9.72 ^a^	51.47 ± 9.69	39.72 ± 3.78 ^b^	44.84 ± 3.92 ^b^
AUC of OGTT (mg/dL*min)	22,170 ± 311.94	27,540 ± 468.09 ^a^	26,047.5 ± 291.98 ^a^	24,147.5 ± 392.73 ^abc^	24,285 ± 488.88 ^ab^

The data are expressed as mean ± SEM. HFD; high-fat diet, Lut50; luteolin (50 mg/kg), Lut100; luteolin (100 mg/kg), Met100; metformin (100 mg/kg), AUC; area under the curve, OGTT; oral glucose tolerance test, HOMA-IR; homeostatic model assessment for insulin resistance. ^a^ *p* < 0.05 vs. control, ^b^ *p* < 0.05 vs. HFD, ^c^ *p* < 0.05 vs. HFD + Lut50 (n = 6/group).

**Table 2:** Effect of luteolin treatment on plasma MDA level, plasma catalase activity, hepatic MDA levels, and hepatic O_2_^•−^ production in HFD-induced MAFLD rats.

Parameters	Control	HFD + Vehicle	HFD + Lut50	HFD + Lut100	HFD + Met100
Plasma:					
Plasma MDA level (μM)	4.15 ± 0.24	8.08 ± 0.29 ^a^	6.97 ± 0.36 ^a^	5.22 ± 0.66 ^b^	5.30 ± 0.77 ^b^
Plasma CAT activity (U/mL)	43.86 ± 7.27	8.22 ± 1.56 ^a^	31.87 ± 4.33 ^b^	35.20 ± 4.39 ^b^	40.94 ± 3.65 ^b^
Liver tissue:					
Hepatic MDA level (nmol/g protein)	360.58 ± 10.48	707.33 ± 34.93 ^a^	548.98 ± 69.52	394.07 ± 24.96 ^b^	397.73 ± 18.09 ^b^
Hepatic O_2_^•−^ production (count/mg dry weight/min)	161.15 ± 24.24	573.92 ± 73.16 ^a^	467.36 ± 104.08	206.69 ± 32.68 ^b^	132.59 ± 56.18 ^b^

The data are expressed as mean ± SEM. HFD; high-fat diet, Lut50; luteolin (50 mg/kg), Lut100; luteolin (100 mg/kg), Met100; metformin (100 mg/kg), MDA; malondialdehyde, CAT; catalase, and O_2_^•−^; superoxide. ^a^ *p* < 0.05 vs. control, ^b^ *p* < 0.05 vs. HFD, (n = 6/group).

## Data Availability

The datasets used and analyzed during the current study are available from the corresponding author on reasonable request.

## Abbreviations

The following abbreviations are used in this manuscript:

MAFLD	Metabolic-dysfunction-associated fatty liver disease
HFD	High-fat diet
AdipoR1	Adiponectin receptor 1
AMPK	AMP-activated protein kinase
PPARγ	Peroxisome proliferator-activated receptor type γ
Lut	Luteolin
Met	Metformin
